# The Limitations of Surgery

**Published:** 1907-01-19

**Authors:** 


The Hospital

A Journal of

The Medical Sciences and Hospital Administration.

Vol. XLI.—No. 1,062. SATURDAY, JANUARY 19, 1907.

### The Limitations of Surgery.

In the thirty-fifth book of his Natural History Pliny tells the story of Apelles and the cobbler where the painter made the stinging retort, "Ne sutor supra crepidam" to the son of Crispin's criticism. The retort passed into a proverb, and the proverb at the present time is in urgent need of application in connection with the popular treatment of medical subjects. The amazing rubbish which the editors of substantial newspapers allow their subordinates to write and publish upon medical topics is almost beyond belief. Compulsory education has taught an enormous number of people in this country to read and write, but it has not yet gone far enough to teach them to think, or even to exercise such critical faculties as they may naturally possess. A loose statement, or the hyperbole of an eminent man is published with as little comment, and often with greater display, than the most accurate and closely reasoned utterances. During the past week the *Daily Chronicle* has signalised itself in this manner on two occasions. In its foreign correspondence it quotes a saying of Professor Posner of Berlin that in future surgeons will find no difficulty in attaching a decapitated head to the trunk provided the operation be carried out with sufficient rapidity, whilst it predicts that the time will come when surgeons will be able to attach an artificial arm or leg to the body in place of the limb which has been amputated, so that the outlook of "surgical possibilities is enough to make one dizzy."

Such statements are as harmful to the ignorant as they are ridiculous to those who know. They are harmful because they are untrue. An editor of a great daily has many difficulties to surmount, but he should at least be able to control his correspondence. Where, however, editors claim that they have failed to appreciate the falsehood, they are clearly unfit for the responsibilities which attach to their office in a free country like England. They are ridiculous because it is apparent to everyone, who knows even the elements of anatomy and physiology, that no surgical procedure could ever allow of the immediate union of all the complex tissues severed in a beheadal or amputation, and it is only by instant repair that life could be preserved in such cases.

Surgery is strictly limited in its methods and in its results by the fundamental facts of life. To the novice the recent advances in surgery appear stupendous, but to those who know the history of the art, it is apparent, that, all the modern operations of which we are so proud were done by our predecessors—the only difference being that what they did occasionally, and by accident, we do regularly and by design. In the higher animals no organ or complex tissue can be wholly removed from the body, even for a moment, and yet live. The very exceptions are only apparent exceptions and prove the rule. When a piece of bone is grafted into a fracture, when a nerve or tendon is spliced into a nerve or tendon, and when a skin grafting is performed with seemingly good results, it is not the extraneous bone, nerve, tendon or skin which becomes incorporated. These structures serve simply as a scaffolding along which the new tissues grow. They give the line of direction and they may even afford the necessary stimulus, but when they have served their purpose they are carried away piecemeal, and leave not a wrack behind. Surgery, therefore, is not joinery, just as it is not butchery.

Many a good surgeon would make a sorry carpenter or a bad butcher—for surgery demands a very intimate knowledge of the phenomena of life, as they are manifested, both, in health and disease. The work of John Hunter and his pupils, long ago, showed, how far removed is the science from the art of surgery. The art of surgery was carried to an acme by Cheselden, and many a one besides, but they had no science. It was left for Hunter to show that scientific surgery depended directly upon pathology, and for the Hunterian school, pathology was morbid anatomy and experimental physiology. The surgeon, who best knows the course taken by disease, obtains the most satisfactory results in his practice. He alone can forecast and, by forecasting correctly, can anticipate, complications. A knowledge of morbid anatomy was, therefore, of immense advantage to surgeons. At a later time, bacteriology developed as a branch of pathology, and the hand of the surgeon was still further strengthened, for he was then able to become prophylactic, and by recognising the cause of his failures to avoid them. By these means, surgery has attained its present position, and there is reason to think, that, it can still advance along many paths, but until the secret of life is disclosed, as a result of work proceeding from the physical and chemical laboratories of the world, the limitations of surgery are definite and final.

